# Trypanocidal activity of ethanolic extracts of *Commiphora swynnertonii* Burtt on *Trypanosoma congolense*

**DOI:** 10.1186/s12906-016-1191-0

**Published:** 2016-07-08

**Authors:** Yakob P. Nagagi, Richard S. Silayo, Eliningaya J. Kweka

**Affiliations:** Department of Veterinary Microbiology and Parasitology, Faculty of Veterinary Medicine, Sokoine University of Agriculture, P. O. Box 3019, CHUO KIKUU, Morogoro, Tanzania; Division of Livestock and Human Diseases Vector Control, Tropical Pesticides Research Institute, P.O. Box 3024, Arusha, Tanzania; Department of Medical Parasitology and Entomology, Catholic University of Health and Allied Sciences, P.O. Box 1464, Mwanza, Tanzania

**Keywords:** Trypanocidal activity, *Commiphora swynnertonii*, *Trypanosoma congolense*

## Abstract

**Background:**

African trypanosomosis is the disease caused by extracellular protozoan parasites of the genus *Trypanosoma* transmitted by tsetse flies. The current study has evaluated the trypanocidal activity of *Commiphora swynnertonii* extracts on *Trypanosoma congolense*.

**Methods:**

The effect of ethanolic stem bark and resinous extracts on motility of *T. congolense* was evaluated by *in vitro* study at concentrations of 2 mg/ml and 4 mg/ml. Then, trypanocidal activity was evaluated by drug incubation infectivity test using mice at concentrations of 0.4 mg/ml and 2 mg/ml. In both studies negative (without drug) and positive (diminazene diaceturate) controls were used.

**Results:**

The *in vitro* study showed that, ethanolic stem bark extract of *C. swynnertonii* at concentration of 4 mg/ml caused complete cessation of motility for *T. congolense* in 30 min. However, resinous ethanolic extract had delayed effect on cessation of motility of *T. congolense* observed at 90 and 100 min post-incubation at concentrations of 4 mg/ml and 2 mg/ml respectively. The drug incubation infectivity test study depicted that ethanolic stem bark extract at concentration of 2 mg/ml significantly (*p =* 0.000) reduced the infectivity of *T. congolense* in mice. However, it did not vary significantly (*P* =0.897) with group treated with diminazene diaceturate incubated mixture.

**Conclusion:**

The current study has provided evidence that, ethanolic stem bark extract of *C. swynnertonii* possess trypanocidal activity against *T. congolense*. Based on these findings, further studies are recommended to determine its potential as a lead to trypanocidal drug discovery.

## Background

African trypanosomosis also known as “sleeping sickness” in human or “nagana” in animals is caused by extracellular protozoan parasites of the genus *Trypanosoma* transmitted by tsetse flies [1]. While nagana has an appreciable contribution to low livestock productivity in rural areas [2], sleeping sickness is disabling and fatal disease that has however remained a neglected tropical disease contributing to rural underdevelopment [3–5]. About 70 million people distributed in approximately 1.55 million km^2^ are at various risk levels of acquiring sleeping sickness in Africa [6]. On the other hand, nagana is known to reduce income accrued to livestock production up to 50 % and is distributed in approximately 10 million km^2^ in Africa leaving many in abject poverty [2, 7]. In Tanzania, nagana is second to East Coast Fever (ECF) in causing cattle mortalities [8].

Nagana has been mainly controlled by the use of chemotherapy. However, chemotherapeutic options are very limited and currently available drugs for use are diminazene diaceturate and isometamidium chloride [9]. The later is a molecule developed by combining diminazene and homidium [10].

Plants have been the source of most active medical compounds for decades in Africa. *Commiphora swynnertonii* (family *Burseracea*) is one of the plant species reported to possess a multifaceted range of ethnobotanical use amongst Dorobo (sub-ethnic group of Masaai tribe) people in Tanzania [11]. It has been claimed to be used for treatment of sexually transmitted diseases, ulcers and wounds (cut wounds and burn wounds), recalcitrant ulcers, abscesses, swelling of legs, chesty cough and scabies [11]. Its resinous exudates are used for treatment of worm infestation and dental caries, cleansing bladder and control of insects such as ticks, lice, bed bugs and mange mites [11]. In fact, previous studies have established plenitude of knowledge about *C. swynnertonii* that have provided pragmatic documents to support its traditional use. Bakari and others [12] reported that resin and root ethanolic extract possess strong anti-microbial activity against *Streptococcus pyogenes*, *Escherichia coli*, *Bacillus subtilis* and *Candida albicans*. Chloroform leaf extracts have been shown to possess highest activity against *Vibrio cholera*, *Shigella flexineri* and *Cryptococcus neoformans* [13]. On the other hand, resinous ethanolic extract has also been shown to possess strong antiviral activity against Newcastle disease virus [14, 15], and anticoccidial activity on *Eimeria* species in chicken [16]. Additionally, essential oils of *C. swynnertonii* leaves have been shown to possess repellent effect on *Rhipicephalus appendiculutus* whereas the hexane and ethyl acetate bark extracts were reported to induce mortality to both nymphs and adults of *R. appendiculatus* [17, 18]. Notably, there is also a recent confirmation of strong acaricidal activity possessed by *C. swynnertonii* exudates [19].

Plant extracts have been found to have antitrypanosomal activity against trypanosomes in different plant extracts evaluated *in vitro* [20]. Considering the previous reports on *C. swynnertonii* extracts activity, there is none which directly underpins the basis of our current study. The basis of this study was; (i) information on its anticoccidial activity against *Eimeri*a species which are protozoan parasites, (ii) evidence of *in vitro* trypanocidal activity of *C. kerstingii* in Nigeria [21] and (iii) hypothesis which was developed based on the literature evidence that sesquiterpenes are among the phytochemical compounds in *Commiphora* species [22] with various forms reported to possess trypanocidal activity *in vitro* and in vivo [23]. Therefore, this study presents the report on the evaluation of extracts of *Commiphora swynnertonii* Burtt against *Trypanosoma congolense* by *in vitro* and drug incubation infectivity test.

## Methods

### Plant materials

Stem pieces and resin of *Commiphora swynnertonii* were collected from Kitwai A village (4°05′42.00 ˝ S and 36°33′34.42 ˝ E), Simanjiro district which is in the northern part of Tanzania. The plant specimen was submitted to National Herbarium of Tanzania, Tropical Pesticides Research Institute, Arusha (Specimen voucher Number CS..01). Confirmation was done by Mr. Emmanuel Mboya and plant specimen deposited at the herbarium. The collected plant materials were transported to Sokoine University of Agriculture for preparation, extraction and *in vitro* and in vivo testing.

### Plant extract preparation

The resin was stored in a refrigerator at 4 °C while the stem bark peeled off and dried under shade for about 4 weeks. Dried bark was ground to fine powder using laboratory mill and stored in an airtight bag in cool dry room until used. Two hundred gram (200 g) of the ground stem bark was weighed and soaked in 400 ml of 99.9 % ethanol in a conical flask sealed with aluminium foil and left for 72 h in a dark place with occasional stirring after which it was first filtered using a piece of cotton wool in a funnel into a conical flask and then using Whatmann® filter paper No. 1. The obtained filtrate was put in beaker and concentrated under ceiling fan at room temperature. The resin was treated differently. After soaking, the ethanol was immediately evaporated at room temperature under ceiling fan. The resulting crude extracts were then stored at 4 °C in airtight bottles until used.

Two (2) grams of both resinous and stem bark extracts were weighed in a bijou bottle separately and each diluted with 10 mls of phosphate buffered saline with glucose (PBSG) (pH, 8.0) to make 200 mg/ml stock solution. Two other extract concentrations (10 mg/ml and 20 mg/ml) were prepared from stock solutions by serial dilutions using PBSG. The extract solutions were prepared just before use and labeled accordingly while the remaining stock solutions stored in a refrigerator at 4 °C until required.

### Trypanosome stock

The trypanosome stock used in this study was a stabilate of putative drug sensitive strain *Trypanosoma congolense* originally isolated from Mikese, Morogoro. This strain is maintained by serial passage in Swiss albino mice at the Small Animal Unit of the Faculty of Veterinary Medicine, Sokoine University of Agriculture. Trypanosomes used to undertake the studies were from mice detected parasitaemic (1.26–2.51 x 10^8^ trypanosomes per milliliter of blood) 4–5 days post inoculation.

### Determination of parasitic load

The parasitic load in mice was monitored in blood from the tail, pre-sterilized with methylated spirit. The number of parasites per milliliter was estimated microscopically at x400 magnification as per Herbert and Lumsden [24]. This involved counting of parasites per field in pure blood or blood diluted with buffered phosphate saline with glucose (pH, 8.0). Logarithm values of these counts were obtained by matching with table of Herbert and Lumsden [24] and converted to antilog to provide absolute number of trypanosomes per milliliter of blood.

### *In vitro* test for trypanocidal activity

In assessing the *in vitro* anti-trypanosomal activity, 5 μl from the prepared stock resinous and stem bark extracts (10 mg/ml and 20 mg/ml) were drawn and poured into separate labeled eppendorf tubes. For reference purposes, three other eppendorf tubes were included, one with 5 μl of PBSG without the plant extract and the other containing 5 μl of 10 mg/ml and 20 mg/ml diminazene diaceturate (Veriben®, Ceva Santé Animale, France). Test *T. congolense*–infected blood was collected from donor Swiss albino mice and diluted using PBSG to make estimated parasitic load of 3.16 x 10^7^ trypanosomes per milliliter from which 20 μl were drawn and poured into the labeled eppendorf tubes including negative (PBSG only) and positive (with diminazene aceturate) control to make effective concentrations of 2 mg/ml and 4 mg/ml of resinous, stem bark extracts and veriben® respectively. These were gently mixed and allowed to stand in an incubator set at temperature of 37 °C for 20 min. Thereafter, about 2 μl of the test mixture were placed on separate microscope slide and covered with cover slips and parasite observed after every 5 min for motility. Cessation or reduction in motility of the parasites in extract treated blood compared to that of the parasite loaded control blood without extract was taken as a measure of trypanocidal activity [25, 26].

### Drug incubation infectivity test

#### Experimental animals

Random-bred male and female, Swiss albino mice 2–4 months old, weighing 25–40 g were used in carrying out the drug incubation infectivity test (DIIT). They were randomly put into six groups kept in plastic cages with wood shavings as beddings and identified with picric acid markings. They were fed with broiler mash (finisher) and tap water provided adequately.

### Reconstitution and incubation

Resinous and stem bark extract concentrations of 20 mg/ml and 100 mg/ml respectively were reconstituted from their 200 mg/ml stock solutions using PBSG. Diminazene diaceturate at 20 mg/ml to be used as treatment control was prepared from commercially available sachet (Veriben®, Ceva Santé Animale, France) using distilled water. Six eppendorf tubes were taken and labeled as G1, G2, G3, G4, G5 and G6. Thereafter, 10 μl from the reconstituted preparations with amount of resinous extract per ml being 20, 100 mg were put in eppendorf tubes G1, G2 respectively; stem bark extract 20, 100 mg in eppendorf tubes G3, G4 respectively; diminazene diaceturate 20 mg/ml in G5 while into tube G6 was put 10 μl of PBSG. This was followed by addition of 490 μl of diluted mouse blood containing 1.58 x 10^7^ trypanosomes per milliliter prepared by diluting blood from donor mouse with PBSG at dilution rate of 1:8 and parasite concentration estimated by the method of Hubert and Lumsden [24]. Gentle mixing was done and incubated at 37 °C for 30 min.

### Study design

Six groups each with five mice were inoculated intraperitoneally with 0.08 mls of incubation mixture (containing extract, diminazene diaceturate or PBSG and trypanosome suspension) as shown in Table 1. For logistical reasons the recorded incubation time varied from 36–56 min. Post inoculation monitoring of mice for parasitaemia by wet smear examination was carried out daily in the first week and thereafter 2–3 times a week for 7 weeks. At least 20 high power fields (x 400) were examined before considering a sample negative.

**Table 1:** Experimental groups, the type of treatment and the time elapsed before inoculation into mice

Group (G)	Extract	Concentration	Incubation Period (Min.)
1	Resinous	0.4 mg/ml	48
2		2 mg/ml	44
3	Stem Bark	0.4 mg/ml	36
4		2 mg/ml	52
5	Diminazene diaceturate	0.4 mg/ml	40
6	PBSG (control)	-	56

### Data analysis

The infection rate post inoculation for the drug incubation infectivity test experiment was determined. The least significance difference (LSD = 0.05) was obtained by one way analysis of variance (ANOVA) using statistical package for social science (SPSS) version 16 (Chicago, SPSS Inc., USA).

## Results

The motility of *T. congolense* after *in vitro* incubation with resinous and stem bark ethanolic extracts of *Commiphora swynnertonii* at concentration of 2 mg/ml and 4 mg/ml is shown in (Table 2). In this study, ethanolic stem bark extract at the concentration of 4 mg/ml caused complete cessation of the motility of *T. congolense* in 30 min. Meanwhile, at the concentration of 2 mg/ml, the trypanosomes were less motile. Complete cessation of motility was observed after 60 min of incubation. In contrast, the resinous extract at comparable concentrations as the stem bark imposed a minimal effect on trypanosomes. This varied from motile to less motile until complete cessation of motility was observed at 90 and 100 min for the concentrations of 2 mg/ml and 4 mg/ml respectively. Diminazene diaceturate imposed less motility on the trypanosomes, complete cessation of motility was observed at 30 and 40 min at concentrations of 4 mg/ml and 2 mg/ml respectively. The trypanosomes however were very motile to being motile until towards the end of the follow up at 100 min in the negative control.

**Table 2:** Motility of *T. congolense* after *in vitro* incubation with various concentrations of *C. swynnertonii* extracts

Test Mixture	Post Incubation follow up (minutes)								
	25	30	40	50	60	70	80	90	100
Resinous 2 mg/ml	+++	++	++	++	++	++	+	+	-
4 mg/ml	++	++	+	+	+	+	+	-	-
Stem Bark 2 mg/ml	++	+	+	+	-	-	-	-	-
4 mg/ml	+	-	-	-	-	-	-	-	-
Veriben 2 mg/ml	+	+	-	-	-	-	-	-	-
4 mg/ml	+	-	-	-	-	-	-	-	-
PBSG (−ve control)	+++	+++	+++	+++	+++	+++	+++	++	++

Key: +++ Very motile, ++ Motile, + Less motile, − None motile

Results for *in vitro* trypanosomes drug incubation, followed by an assessment of infectivity to mice are summarized in Table 3. All the mice in group 1 were found to be infected at day 4 post inoculation. There was variation in infectivity of trypanosomes in mice in group 2. The infection was seen on 3rd day whereas in day seven, all the mice were infected. In group 3, infection was seen on the 2nd day and all the mice were found infected on day 4. Group 4 had one mouse infected at day 9 while the rest remained trypanosome free during the entire period of the experiment. None of the mice in group 5 had the infection. In group 6, infection was detected on the 2nd day and all the mice were infected on day 4. Nevertheless, infectivity of *T. congolense* in G4 was reduced significantly (*p =* 0.000) with respect to G6 (group inoculated with infective diluted blood without drug) (Table 4). However, there was no significance difference (*p =* 0.897) between G4 and G5.

**Table 3:** Proportions of mice positive for *T. congolense* Mikese parasitaemia after incubation with *C. swynnertonii* extracts

	Groups inoculated					
	Number of mice infected/total mice present in the group					
Days post inoculation	G1 (resinous, 0.4 mg/ml)	G2 (resinous, 2 mg/ml)	G3 (bark, 0.4 mg/ml)	G4 (bark, 2 mg/ml)	G5 (dimin. diacet., 0.4 mg/ml)	G6 (PBSG, drug free)
0	0/5	0/5	0/5	0/5	0/5	0/5
2	0/5	0/5	1/5	0/5	0/5	1/5
3	0/5	1/5	3/5	0/5	0/5	2/5
4	5/5	2/5	5/5	0/5	0/5	5/5
6	5/5	4/5	5/5	0/5	0/5	4/4
7	5/5	5/5	3/3	0/5	0/5	2/2
8	4/4	5/5	2/2	0/5	0/5	1/1
9	4/4	5/5	1/1	1/5	0/5	1/1
14	3/3	4/4	1/1	0/4	0/5	1/1
35	2/2	2/2	1/1	0/4	0/4	1/1
45	1/1	1/1	1/1	0/4	0/4	1/1
53	0	1/1	1/1	0/4	0/4	0

**Table 4:** Infectivity of *T. congolense* in mice following drug incubation infectivity test of *C. swynnertonii* extract

(I)	(J)	Mean			95 % Confidence Interval	
Groups treated	Groups treated	Difference (I – J)	Standard Error	Significance level	Lower Bound	Upper Bound
Groups treated	Groups treated	Difference (I – J)	Standard Error	Significance level	Lower Bound	Upper Bound
G4	G5	.02222	.17021	.897	–.3200-	.3645
	G6	–.71111	.17021	.000	–1.0533	–.3689

## Discussion

The findings of this study have demonstrated that, the stem bark extracts at concentration of 4 mg/ml caused complete cessation of motility of *T. congolense* within 30 min in the *in vitro* study of the ethanolic extracts of *C. swynnertonii*. This observation partly matches with that of a previous study whereby methanolic stem bark extract of *C. kerstingii* at concentrations of 2 and 4 mg/ml were both shown to cause complete cessation of motility on *T. brucei brucei* within 30 min. [21] The slight difference observed might be attributed to biochemical variations within species, geographical location, methods or mode of extraction, solvent used [27], and the season at which the plant material harvested [28]. On the other hand, the resinous extract had lower observed effect on the motility of *T. congolense* with cessation of motility observed at 90 and 100 min post incubation at concentrations of 4 and 2 mg/ml respectively. Among the extracts of *C. swynnertonii* previously studied, resinous extract was shown to possess strong antimicrobial activity compared with stem bark, root bark and leaves extracts [29]. In contrast, this study has found that the stem bark extracts exhibit more *in vitro* antitrypanosomal activity than the resinous extract. A study conducted from 37 extracts in Tanzania have reported that, stem bark extracts of *C. emenii* possess strong *in vitro* antitrypanosomal activity relative to the other plant parts (leaves and root bark) [30].

Drug incubation infectivity tests showed that after incubation with mixture of ethanolic stem bark extract at concentration of 2 mg/ml for 52 min the reduction of infectivity of *T. congolense* Mikese compared to negative control was highly significant (*P* = 0.000). However, it did not vary significantly (*P* =0.897) with group treated with diminazene diaceturate incubated mixture. Therefore, from drug incubation infectivity test experiment it was confirmed that the stem bark extracts of *C. swynnertonii* possess trypanocidal activity while resinous extract had no activity at similar concentrations. However, our current study should never be extrapolated to mean efficacy in vivo due to metabolic processes which occur in multicellular organisms. Both techniques used in this study, exposed the trypanosomes to a specified drug concentration, thereby avoiding potential problems such as drug distribution, drug half-life and bioavailability of the drug, and the behavior exhibited by the trypanosomes in the host. [24].

The current study has added *C. swynnertonii* Burtt to the list of plant species of similar genus (genus *Commiphora*) that were previously tested *in vitro* and found to possess antitrypanosomal activity. Other plant species included are *C. kerstingii* Engl. in Nigeria [21], and *C. emenii* Engl. in Tanzania [30]. The results of this study concur with the assumptions made by Freiburghaus and others that, antitrypanosomal effects might be due to phytochemical compounds universally existing in a plant genus [30]. These include tannins, phlobatannins, terpenoids, flavonoids, cardiac glycosides, steroids, saponins and anthraquinones which were confirmed to be contained in *C. swynnertonii* by Bakari [29]. Nevertheless, the trypanocidal principles of the species previously tested and the one tested in this study are still unknown. Although phytochemical analysis of various species of *Commiphora* have shown sesquiterpenes, a diverse group of terpenoids as among the chemical compounds [22], with various forms of sesquiterpenes reported to possess trypanocidal activity *in vitro* and in vivo [23], interpretation of our results based on the biochemical compound(s) responsible for the valid trypanocidal activity from genus *Commiphora* awaits further studies using selected biochemical components.

## Conclusion

The current study has provided the first indication that ethanolic bark extracts of *C. swynnertonii* possess trypanocidal activity. Considering the two extracts (stem bark and resinous) of *C. swynnertonii* evaluated in this study, the phytochemical compound(s) responsible for antitrypanosomal activity may be well placed in the stem bark extract than in resinous extract. Therefore, studies are in progress to determine the potential of stem bark extract of *C. swynnertonii* as a trypanocidal drug and or alternative source of lead compound(s) for trypanocidal drug discovery.

## Abbreviations

DIIT, Drug Incubation Infectivity Test; PBSG, Phosphate buffered saline with glucose
